# Molecular Mechanisms of *Candida albicans* Adhesion to Maxillofacial Silicone Elastomers: Integrating Candidate Molecular Pathways with Material Surface Properties

**DOI:** 10.3390/ijms27209031

**Published:** 2026-10-12

**Authors:** Ahmed M. Hamarsha, Mustafa Atatra, Nezar Watted, Ahmad Adebayo Irekeola, Mohammad Majduddin Sulaiman, Nurulezah Hasbullah

**Affiliations:** 1 Department of Dentistry, Arab American University, Jenin P.O. Box 240, Palestine; ahmad.hamarsha@aaup.edu or ahmed.hamarsha@student.usm.my (A.M.H.); mustafa.atatra@aaup.edu or mustafa.atatra@student.usm.my (M.A.); nezar.watted@aaup.edu or prof.watted@gmx.net (N.W.); 2 School of Dental Sciences, Universiti Sains Malaysia, Health Campus, Kubang Kerian 16150, Malaysia; irekeola@usm.my (A.A.I.); mmajduddin@usm.my (M.M.S.); 3 University Hospital of Würzburg, Julius-Maximilians-University of Würzburg, 97070 Würzburg, Germany; 4 Microbiology Unit, Department of Biological Sciences, College of Natural and Applied Sciences, Summit University Offa, Offa PMB 4412, Kwara State, Nigeria

**Keywords:** acquired salivary pellicle, adhesins, antifungal target engagement, biofilm regulatory network, *Candida albicans*, maxillofacial silicone, mechanotransduction, thigmotropism

## Abstract

Colonisation of maxillofacial prostheses by *Candida albicans* is conventionally attributed to surface roughness, porosity, and wettability properties that explain where cells accumulate rather than why particular surfaces promote biofilm formation. This review reinterprets the in vitro literature on *C. albicans* adhesion to maxillofacial silicone elastomers through the underlying molecular events. Nine eligible studies identified through searches of PubMed, Scopus, and ScienceDirect conducted on 31 March 2026 were appraised with the QUIN tool and mapped onto a six-stage molecular cascade: surface conditioning, reversible attachment, adhesin engagement, morphogenesis, matrix assembly, and antifungal tolerance. Three conclusions emerged. First, *C. albicans* senses topography through mechanically gated calcium channels and converts mechanical contact into hyphal commitment; roughness is therefore a morphogenetic cue that surface finishing can withhold. Second, the tested agents—silver nanoparticles, chitosan, thymol, and carvacrol, and a *Lactobacillus brevis*-derived biosurfactant—engage distinct molecular targets and are not mechanistically interchangeable. Third, most included studies evaluated silicone without salivary conditioning, whereas the clinical interface is an acquired salivary pellicle that supplies both adhesion receptors and antimicrobial peptides. All studies fell within the medium risk-of-bias band (50.0–62.5%). Pellicle-conditioned models and molecular readouts are required. The mechanistic interpretations discussed in this review are derived from the broader *C. albicans* literature and have not been directly demonstrated on maxillofacial silicone elastomers.

## 1. Introduction

Maxillofacial prostheses restore facial form and function after congenital malformation, trauma, or oncological resection, and silicone elastomers remain the materials of choice for their compliance, chemical stability, and capacity to reproduce the optical and tactile qualities of skin [1,2]. Their principal biological failure mode is microbial colonisation, and *Candida albicans* is the organism most consistently recovered from prosthetic surfaces [3,4]. The prosthodontic literature has characterised this problem in materials terms. Surface roughness, porosity, and wettability are repeatedly identified as determinants of microbial accumulation on dental materials [5,6], an association shown for *Candida* on maxillofacial silicone by the studies appraised in this review, and interventions are evaluated by the reduction in colony counts they produce. This framing has been productive, but it is descriptive rather than mechanistic. It establishes that rougher surfaces accumulate more organisms without explaining what a rough surface does to the organism, and it treats chemically unrelated interventions as interchangeable members of a single category of “antimicrobial modification” on the basis of a shared endpoint.

Fungal cell biology addresses precisely these questions, though the two literatures rarely meet [7]. Adhesion to abiotic substrates is mediated by defined cell-surface glycoproteins whose structural basis is now resolved [8,9]. Commitment to the hyphal form, which supplies the mechanical scaffold of a mature biofilm, is governed by an interconnected network of transcriptional regulators [10,11]. Tolerance to antifungal agents within a biofilm arises from phase-specific mechanisms that shift from efflux-mediated to sterol-mediated as the community matures [12]. And, most directly relevant here, *C. albicans* possesses a characterised mechanosensory apparatus through which it detects and responds to surface topography [13,14].

Despite advances in fungal cell biology, three important gaps remain. First, the relationship between silicone surface characteristics and *C. albicans* adhesion has largely been described as a material-level association, with limited integration of the molecular events that may link surface topography to fungal adhesion and morphogenesis. Second, antimicrobial strategies applied to maxillofacial silicone have generally been compared according to their reported antifungal efficacy, although chemically distinct interventions may act at different stages of colonisation and biofilm development. Third, the prevailing experimental model largely examines *C. albicans* interaction with unconditioned silicone, whereas prosthetic surfaces in clinical use are exposed to saliva and rapidly acquire a conditioning film that may alter the biological interface. These gaps limit mechanistic interpretation and reduce the translational relevance of the existing evidence.

Accordingly, this review maps the available in vitro evidence on *C. albicans* adhesion and antimicrobial strategies involving maxillofacial silicone elastomers and interprets the findings against a molecular framework derived from the broader *C. albicans* literature. Three aims follow: first, to examine the evidence underlying the relationship between silicone surface characteristics and fungal adhesion; second, to distinguish antimicrobial strategies tested on these materials according to their proposed biological point of action rather than solely by reported efficacy; and third, to identify where the prevailing experimental model departs from the biological conditions encountered by maxillofacial prostheses in clinical use.

## 2. Materials and Methods

### 2.1. Protocol and Reporting

This review was conducted in accordance with the methodological framework for reviews proposed by Arksey and O’Malley [15] and reported according to the Preferred Reporting Items for Systematic Reviews and Meta-Analyses extension for Scoping Reviews (PRISMA-ScR) [16]. The completed PRISMA-ScR checklist is provided as Supplementary Table S2. The review protocol was registered on the Open Science Framework (OSF; https://doi.org/10.17605/OSF.IO/5S4WG; registered on 15 April 2026).

### 2.2. Review Question and Conceptual Framework

The review question was structured using the Population–Concept–Context framework recommended for reviews. The population was *Candida albicans;* the concept was adhesion and subsequent biofilm formation; and the context was recognised maxillofacial silicone elastomers evaluated under in vitro conditions. The question addressed was: what material properties and antimicrobial modifications influence *Candida albicans* adhesion to maxillofacial silicone elastomers, and by what molecular mechanisms do these effects operate?

### 2.3. Information Sources and Search Strategy

Three electronic databases were searched: PubMed (National Library of Medicine, Bethesda, MD, USA), Scopus (Elsevier, Amsterdam, The Netherlands), and ScienceDirect (Elsevier, Amsterdam, The Netherlands). The final searches were conducted on 31 March 2026.

Search terms combined controlled vocabulary and free-text terms across three concept blocks, joined with the Boolean operator AND, with terms within each block joined by OR. Block 1 (organism): “*Candida albicans”*, *Candida*, fungal. Block 2 (substrate): “maxillofacial silicone”, “silicone elastomer”, “maxillofacial prosthesis”, “facial prosthesis”. Block 3 (outcome or intervention): adhesion, adherence, biofilm, colonisation, antifungal, antimicrobial, nanoparticle. The complete search string as executed in each database is reproduced in Supplementary Table S3. No publication-year, date-range, language, publication-type, or study-design filters were applied at the search stage. Reference lists of all included articles were hand-searched for additional eligible studies.

### 2.4. Eligibility Criteria

Studies were eligible for inclusion if they met all of the following criteria: (i) primary in vitro investigation; (ii) evaluation of a recognised maxillofacial silicone elastomer; (iii) reporting of a quantitative outcome for *Candida* adhesion, biofilm formation, or antifungal activity on that material; and (iv) publication in English in a peer-reviewed journal.

Studies were excluded if they met any of the following criteria: (i) narrative or systematic reviews, conference abstracts, case reports, editorials, or letters; (ii) evaluation of materials other than maxillofacial silicone elastomers, including denture base acrylics and hard-relining materials, unless a maxillofacial elastomer was assessed as a separate arm; (iii) reporting of qualitative or purely descriptive outcomes without quantification; (iv) evaluation of microorganisms other than *Candida* species without a *Candida* arm; and (v) in vivo or clinical study designs, which were recorded during screening but not carried forward, since the review was concerned with mechanistic evidence obtained under controlled conditions.

### 2.5. Study Selection

**Figure 1:**
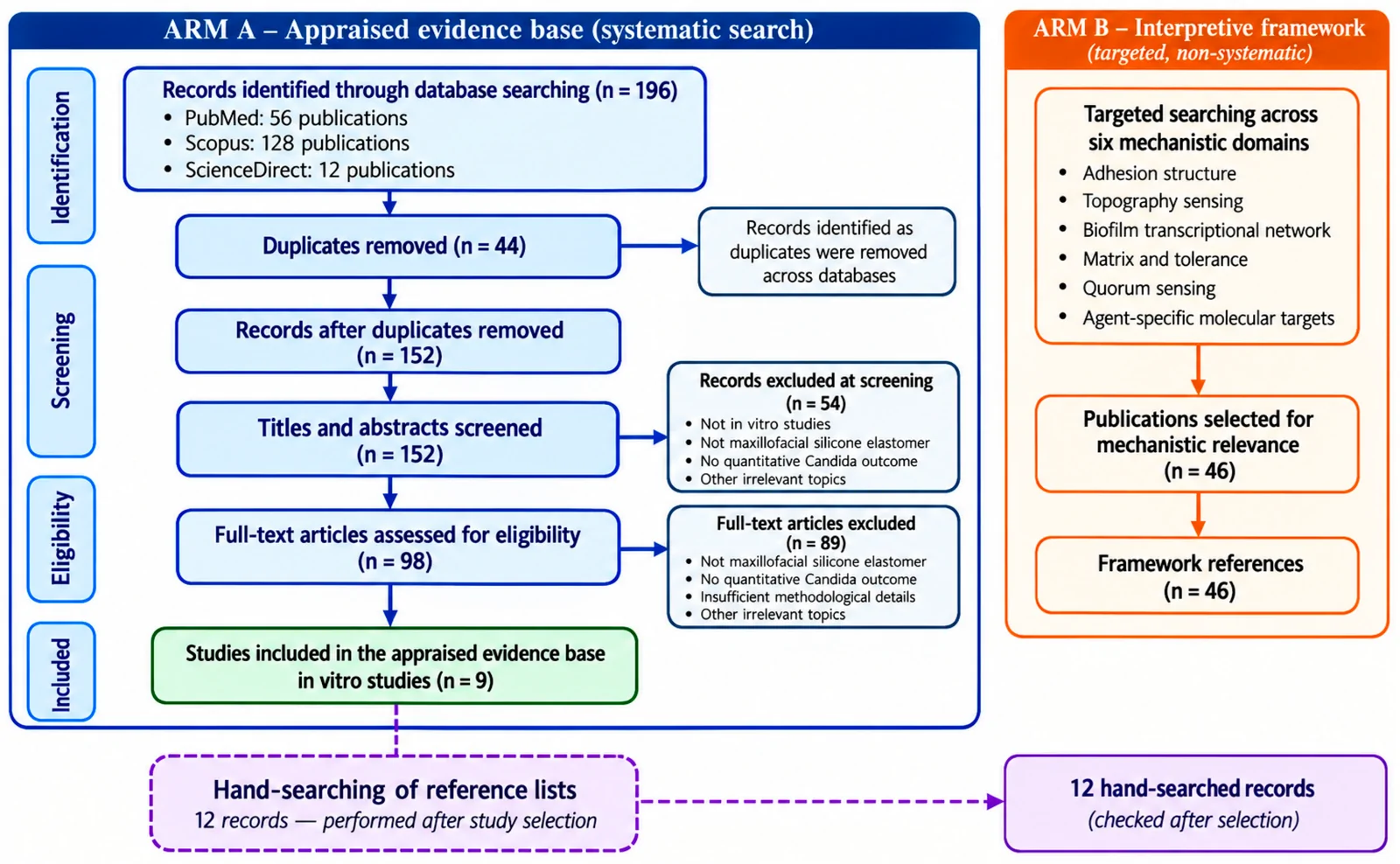
Identification of studies and development of the interpretive framework. Arm A shows systematic identification of the appraised evidence base through database searching of PubMed, Scopus, and ScienceDirect, yielding nine eligible in vitro studies. Arm B shows targeted, non-systematic identification of the mechanistic literature used to interpret those studies, comprising 46 publications selected across six domains. The two arms are reported separately because they were assembled by different methods and serve different functions; this asymmetry is stated in Section 2. Blue boxes and solid arrows represent the systematic study-identification pathway (Arm A); orange boxes and arrows represent the targeted, non-systematic identification of the interpretive framework (Arm B); and purple dashed boxes and arrows represent hand-searching of reference lists conducted after study selection.

Records retrieved from all three databases were exported to a reference manager and deduplicated. Titles and abstracts were screened against the eligibility criteria by two reviewers working independently, and full texts of all potentially eligible records were then retrieved and assessed. Disagreements were resolved through discussion, and where consensus could not be reached, a third reviewer was consulted to achieve final agreement. Reasons for exclusion at the full-text stage were recorded and are reported in Figure 1.

### 2.6. Data Charting

A data charting form was developed a priori and piloted on two included studies before application to the full set. The following variables were extracted from each included study: first author and year of publication; country in which the work was performed; study design; silicone elastomer evaluated, including commercial designation and manufacturer; specimen geometry and number; *Candida* species and strain, and inoculum density; intervention or material factor evaluated, with concentration where applicable; definition of the control condition; incubation conditions and duration; outcome measure and unit; reported surface characterisation, including roughness and contact angle where available; principal quantitative findings with associated statistical significance; and stated limitations. Charting was performed by two reviewers and verified by a third. Where a required datum was not reported, this was recorded as “not stated” rather than inferred.

### 2.7. Critical Appraisal of Individual Sources of Evidence

Methodological quality was appraised using the QUIN tool, an instrument developed specifically for the assessment of risk of bias in in vitro studies in dentistry [17]. The tool comprises twelve criteria: clearly stated aims and objectives; detailed explanation of sample size calculation; detailed explanation of sampling technique; details of the comparison group; detailed explanation of the methodology; operator details; randomisation; method of measurement of outcome; outcome assessor details; blinding; statistical analysis; and presentation of results.

Each criterion was scored as adequately specified (2 points), inadequately specified (1 point), or not specified (0 points). Criteria judged not applicable to a given study were excluded from that study’s denominator. The total score was expressed as a percentage of the maximum attainable, calculated as the sum of points divided by twice the number of applicable criteria, multiplied by 100. Studies scoring above 70% were classified as at low risk of bias, those scoring 50–70% at medium risk, and those scoring below 50% at high risk. Appraisal was performed on the published full texts. Per-criterion scores and the textual basis for each judgement are reported in Supplementary Table S1. No study was excluded on the basis of its appraisal score; the appraisal was used to contextualise the synthesis rather than to weight it.

### 2.8. Synthesis of Results

Outcomes were synthesised narratively. Quantitative pooling was not attempted because the included studies reported non-commensurable outcome measures, comprising colony-forming unit counts, cells per unit area, tetrazolium salt reduction, and inhibition zone diameter, and because control conditions were defined differently between studies. Reductions relative to control are therefore reported as stated by each source study and are interpretable only within that study. Findings were charted against the six-stage molecular cascade described in Section 2.9 and are presented by cascade stage rather than by intervention class.

### 2.9. Identification of the Mechanistic Framework

Because the objective was mechanistic interpretation rather than effect estimation, the charted findings were mapped onto a molecular framework assembled from the wider *Candida* cell-biology literature. Publications supporting this framework were identified by targeted searching of PubMed across six domains: adhesin structure and function; surface topography sensing and mechanotransduction; the biofilm transcriptional regulatory network; biofilm matrix composition and antifungal tolerance; quorum sensing; and the molecular targets of the specific agents evaluated in the included studies. Publications were selected on the basis of mechanistic relevance and were not subject to the systematic screening or appraisal procedures applied to the primary evidence base. This asymmetry is deliberate and is represented in Figure 1: Arm A constitutes the evidence appraised in this review, whereas Arm B provides the interpretive framework. Framework publications are cited as supporting literature and are not treated as evidence concerning maxillofacial silicone elastomers.

## 3. Results

### 3.1. Study Selection and the Molecular Framework

The three database searches retrieved 196 records: 56 from PubMed, 128 from Scopus, and 12 from ScienceDirect. After removal of 44 duplicates, 152 unique records remained for title and abstract screening. Reference-list screening of the included studies subsequently identified 12 additional records. Nine in vitro studies met all eligibility criteria and were included in the review (Figure 1). These evaluated VST-30, VST-50, A-2006, A-2186F, MDX4-4210, M511, Multisil, room-temperature vulcanised maxillofacial silicone, and unspecified medical-grade elastomers and tested intrinsic surface properties, nanoparticle incorporation, surface coating, natural antifungal compounds, and a microbially derived biosurfactant.

Eight of the nine studies reported specimen numbers: 48 (Kurtulmus et al. [18]), 24 (Atay et al. [19]), 35 (Khalaf et al. [20]), 180 (Chong et al. [21]), 80 (Sri et al. [22]), 40 (Ramadhan and Abdul-Ameer [23]), 8 per measurement (Zhou et al. [24]), and 42 (Mat-Rani et al. [25]). Ceresa et al. [26] described assays performed in triplicate and repeated three times but stated no total. No study reported an a priori sample size calculation.

**Figure 2:**
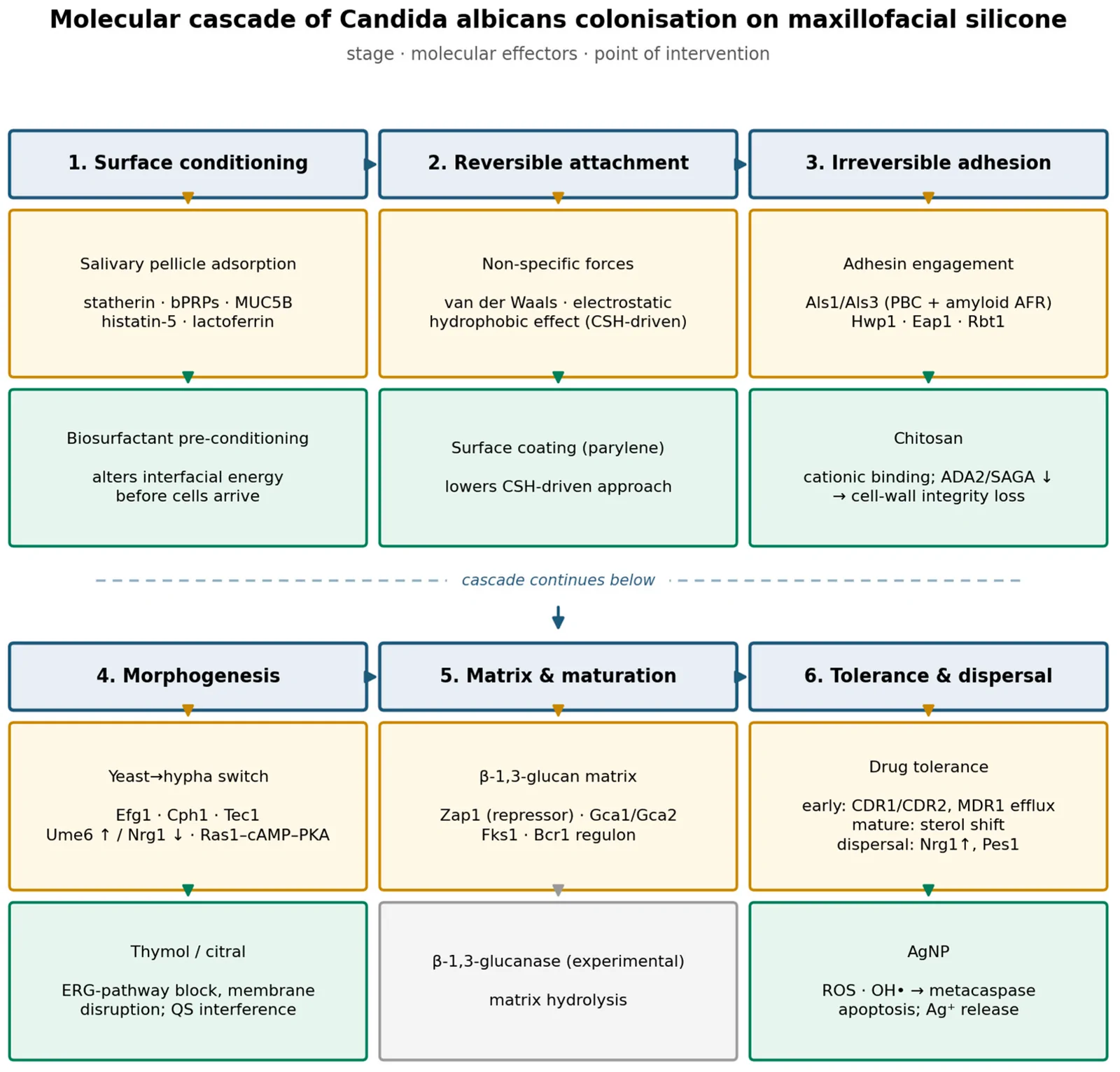
Molecular cascade of *Candida albicans* colonisation on maxillofacial silicone. The upper row gives the six sequential stages of colonisation; the middle row identifies the molecular effectors documented at each stage; the lower row indicates where each intervention class tested on maxillofacial silicone engages the cascade. Agents acting at different stages are not mechanistically equivalent even where their reported reduction in colony counts is similar. The grey panel denotes a matrix-directed strategy supported in the wider literature but not yet evaluated on maxillofacial silicone. CSH, cell-surface hydrophobicity; PBC, peptide-binding cavity; AFR, amyloid-forming region; QS, quorum sensing. Blue boxes represent the six sequential stages of the colonisation cascade, yellow boxes identify the molecular effectors associated with each stage, and green boxes indicate the intervention classes targeting the corresponding stages. Arrows indicate the sequential progression of the cascade and the points at which individual interventions engage it.

Figure 2 sets out the molecular cascade against which these studies are interpreted. It has six stages, and the studies in this field address only a subset of them.

### 3.2. Surface Roughness as a Morphogenetic Cue

Studies investigating intrinsic material properties have consistently reported a positive association between surface roughness and fungal accumulation, as well as between surface porosity and fungal retention [18,19,20]. These associations are commonly attributed to the increased surface area available for microbial attachment and the formation of protective niches that reduce exposure to shear forces. While both mechanisms contribute to fungal accumulation and retention, they do not fully explain the observed relationship.

Quantitative support for this association is thinner than the consistency of the qualitative finding suggests. Only three of the nine included studies reported any measured surface parameter. Khalaf et al. reported a mean roughness of 0.12 ± 0.08 µm for silicone produced from coated gypsum moulds against approximately 0.22 µm for unmodified specimens, with correspondingly lower microbial adhesion (*p* < 0.001) [20]; Atay et al. and Zhou et al. reported contact angles [19,24]. The remaining six studies invoked surface properties without measuring them, so the roughness–adhesion relationship in this literature rests on three quantitative observations. Moreover, these parameters were not characterised in the same way across studies, and they should not be treated as independent factors: roughness, porosity, wettability, and surface chemistry can each influence the others, so the differences in *Candida* adhesion reported between materials cannot always be attributed to any single surface parameter.

*C. albicans* detects surface topography actively. Directional hyphal growth along surface features, termed thigmotropism, is attenuated by stretch-activated ion channel blockers and by L-type calcium channel antagonists, implicating mechanically gated calcium entry in the response [27]. Hyphae framework rather than as experimental evidence specific to this material.

**Figure 3:**
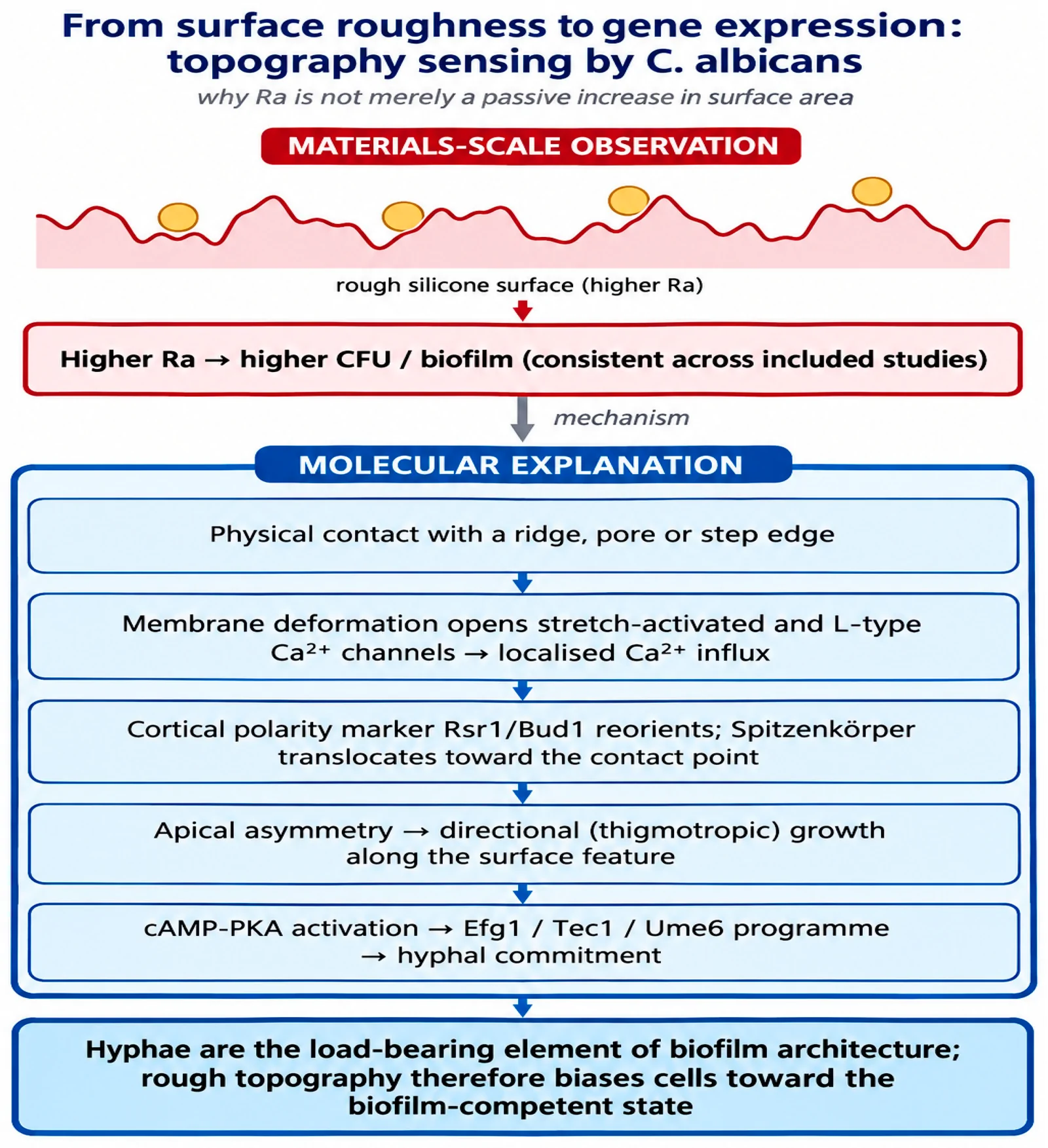
From surface roughness to gene expression. Top: the material-scale association between surface roughness and fungal accumulation reported across the included studies. Bottom: the proposed mechanosensory pathway through which *C. albicans* converts contact with a topographic feature into hyphal commitment, involving mechanically gated calcium entry, reorientation of the Rsr1/Bud1 polarity marker and the Spitzenkörper, and downstream cyclic AMP–protein kinase A signalling. The two sections illustrate the same process at different biological scales. The lower panel depicts a mechanism established on model substrates in the wider C. albicans literature; it has not been demonstrated directly on maxillofacial silicone. Red denotes the materials-scale observations reported across the included studies, whereas blue denotes the proposed molecular mechanism derived from the wider *C. albicans* literature.

Figure 3 sets out the consequence. Hyphal filaments constitute the load-bearing element of biofilm architecture, and the cyclic-AMP–protein kinase A axis that responds to mechanical input also drives the Efg1, Tec1, and Ume6 programme [28,29]. A rough surface therefore does more than retain cells; it biases the cells it retains toward the biofilm-competent state. On this reading, surface finishing withholds a morphogenetic signal rather than merely removing shelter. This reading is a mechanistic interpretation extrapolated from the wider *C. albicans* literature; none of the included studies measured signalling events or gene expression on silicone, so the proposed pathway remains to be tested on this substrate.

Lacking the cortical polarity marker Rsr1/Bud1 leads to failure in responding to topographic features, and on contact, the Spitzenkörper translocates toward the point of contact, producing an apical asymmetry that redirects growth [30]. Directional responses are calcium-dependent [31], and nanoscale topographic structures are sufficient to control the yeast-to-hypha switch itself [32]. It should be emphasised that none of this mechanistic evidence was generated on maxillofacial silicone: the studies cited above used microfabricated or otherwise model substrates, and the link between surface roughness, mechanosensing, and morphogenesis has not been directly demonstrated on these elastomers. It is presented here as an interpretive observation.

A further finding from the coating literature highlights the interaction between surface topography and chemistry. On surfaces bearing microcone topography, *C. albicans* has been shown to survive antifungal surface coatings that are effective on flat substrates [33]. Topography and chemistry are therefore not independent variables, and a coating validated on polished specimens cannot be assumed to perform equivalently on a milled or additively manufactured surface.

### 3.3. Adhesin Engagement at the Elastomer Interface

Attachment to abiotic substrates is mediated by the agglutinin-like sequence (Als) family, comprising eight cell-surface glycoproteins, together with Hwp1, Eap1, and Rbt1 [34]. Structural analysis has localised adhesive function to the N-terminal peptide-binding cavity: strains in which this cavity is disabled are phenotypically indistinguishable from deletion mutants [35]. The separate amyloid-forming region contributes to aggregation rather than primary adhesion [36,37], and under mechanical extension the T domains unfold to expose an amyloidogenic sequence that supports cell-to-cell bonding [38].

This architecture bears directly on the wettability findings. Morphogenesis substantially increases cell-surface hydrophobicity through redistribution and force-induced unfolding of Als adhesins and their associated mannans, exposing hydrophobic groups that were previously buried [39]. Cell-surface hydrophobicity is therefore not a fixed species property against which a material can be matched but a variable that changes as the organism transitions, which in turn is influenced by the surface itself. The relationship between contact angle and adhesion reported by Atay et al. [19] is thus bidirectional rather than a simple material ranking.

### 3.4. Antimicrobial Agents Act at Different Molecular Points

**Figure 4:**
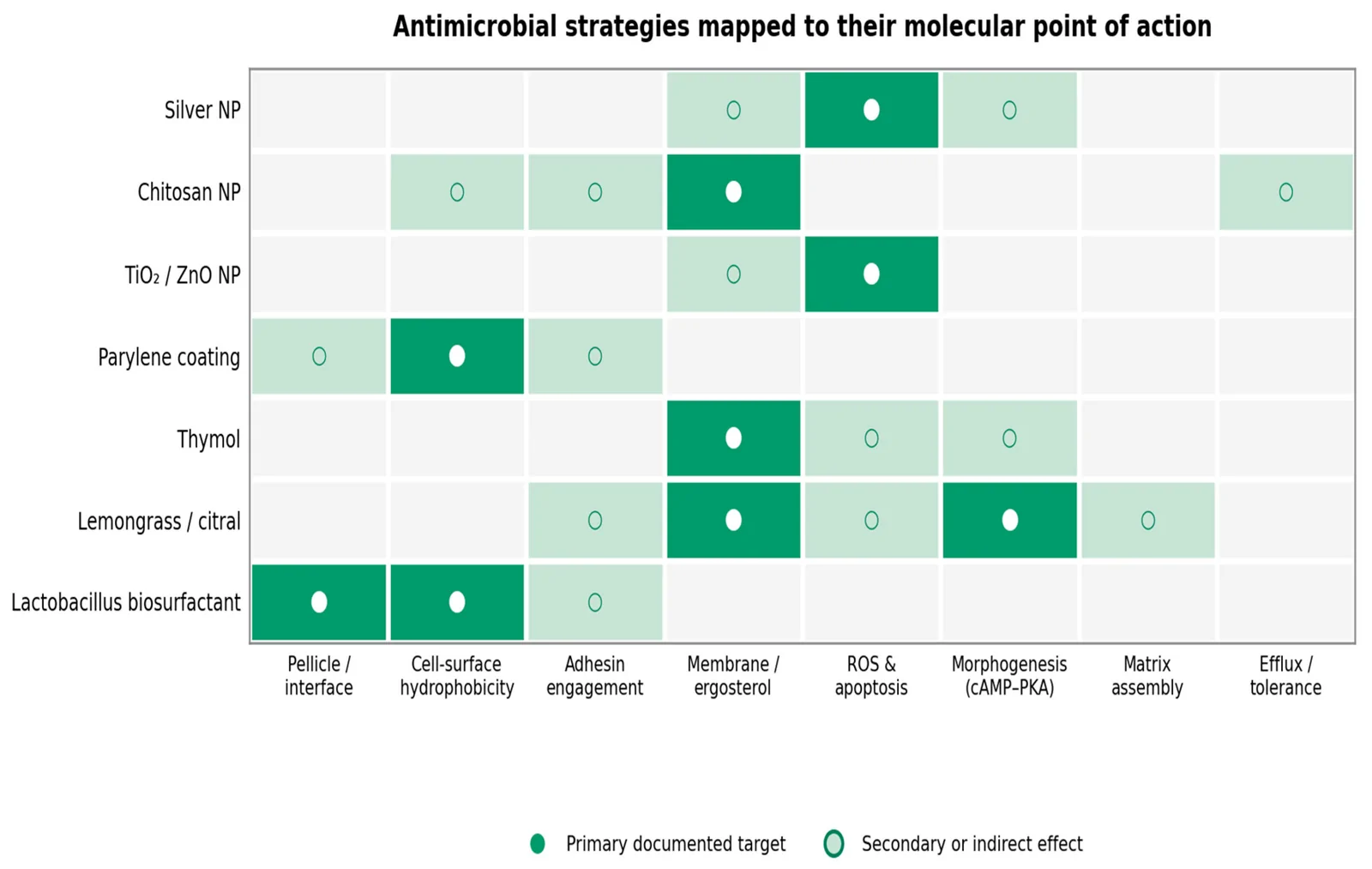
Antimicrobial strategies mapped to their molecular points of action. Dark green indicates a primary documented molecular target, whereas light green indicates a secondary or indirect effect; grey indicates no documented molecular target or effect. These assignments were derived from mechanistic studies in the broader Candida literature rather than from studies of maxillofacial silicone, which did not report molecular-level readouts. The distribution indicates that none of the antimicrobial strategies evaluated to date has been documented to directly target matrix assembly or biofilm-phase tolerance, two stages closely associated with clinical persistence.

The interventions tested on maxillofacial silicone are frequently grouped by outcome. Grouping them by molecular target instead (Figure 4) makes clear that they are not variants of a single approach.

Silver nanoparticles induce a genuine apoptotic programme: exposure elevates hydroxyl radical production, reduces mitochondrial membrane potential, releases cytochrome c, and activates metacaspases with phosphatidylserine externalisation and nuclear fragmentation [40]. Ultrastructural work shows membrane discontinuities and inhibition of budding [41]. This account requires qualification, however, since oxidative-stress-independent toxicity has also been demonstrated [42]; reactive oxygen species are unlikely to be the sole mechanism.

Titanium dioxide and zinc oxide nanoparticles act through comparable oxidative mechanisms, and combined ZnO–TiO_2_ nanostructures generate greater reactive oxygen species production and oxidative stress than either oxide alone. That evidence derives from *Aspergillus flavus* rather than *C. albicans*, and its authors explicitly caution that mechanisms beyond ROS-induced apoptosis remain to be characterised [43]. Neither oxide was evaluated on maxillofacial silicone in any study reviewed here, so their inclusion in Figure 4 reflects mechanistic plausibility rather than direct evidence on this substrate.

Chitosan operates differently. The cationic polymer interacts electrostatically with the anionic cell envelope, producing potassium efflux, altered transmembrane potential, and increased calcium uptake [44]. Chitosan additionally represses ADA2 and the Ada2-dependent cell-wall genes ALS2, PGA45, and ACE2, together with the efflux transporters MDR1 and CDR1 [45]. Cells lacking ADA2 display cell-wall thinning comparable to chitosan-treated wild-type. Chitosan therefore attacks cell-wall integrity at the transcriptional level and simultaneously suppresses two efflux pumps implicated in azole tolerance—a combination that argues for evaluating it alongside conventional antifungals rather than as a standalone agent [46].

A caveat attaches to the chitosan evidence on maxillofacial silicone specifically. Sri et al. quantified the antifungal effect by disk diffusion, reporting mean inhibition zone diameters of 5.28, 10.56, and 17.60 mm at 1.5, 3, and 5 wt%, respectively, against a control value of zero (*p* < 0.0001) [22]. However, interpretation of these findings warrants caution because disk diffusion primarily measures the activity of diffusible antimicrobial substances released from the tested material and does not directly assess microbial adhesion or biofilm formation. Moreover, intact chitosan nanoparticles incorporated within a hydrophobic silicone elastomer would not necessarily be expected to diffuse through the polymer matrix under these assay conditions. Thus, the reported inhibition zones demonstrate that the chitosan-containing elastomer exhibited measurable antifungal activity, but they do not establish nanoparticle diffusion through the silicone matrix or demonstrate a reduction in fungal adhesion or biofilm formation. Thymol and carvacrol disrupt ergosterol biosynthesis and membrane integrity, inhibit plasma-membrane H^+^-ATPase activity, and cause leakage of cellular constituents; unlike the fungistatic azoles, they are fungicidal and retain activity against azole-resistant isolates [47,48]. Essential oils modulate ERG-pathway gene expression directly [49]. The non-monotonic dose response observed by Ramadhan and Abdul-Ameer, in which 0.75 wt% outperformed 1.0 wt% [23], is consistent with a membrane-partitioning agent whose distribution within a polymer matrix need not scale linearly with loading, though this was not investigated.

Biosurfactants act before any of these. As amphiphiles, they condition the interface, rendering it more hydrophilic and displacing the interfacial water arrangement that permits close approach of a hydrophobic cell to a hydrophobic substratum. That cell-free preparations retain full anti-adhesive activity and that planktonic growth is unaffected [26] confirm an interfacial rather than a cytotoxic mechanism and place this class alone at stage one of the cascade.

### 3.5. What the Cascade Shows Is Missing

Two stages of Figure 2 are unaddressed by any agent tested on maxillofacial silicone. No intervention targets matrix assembly, although the β-1,3-glucan matrix is the principal determinant of biofilm drug tolerance. Zap1 negatively regulates soluble β-1,3-glucan through the glucoamylases Gca1 and Gca2 [50]; matrix glucan sequesters fluconazole in an FKS1-dependent manner [51]; and enzymatic degradation of the matrix restores antifungal susceptibility [52]. Nor does any agent address biofilm-phase tolerance, which is itself stage-dependent: efflux pumps CDR1, CDR2, and MDR1 dominate early, whereas altered membrane sterol composition dominates in mature biofilms [53,54].

The clinical implication is that agents concentrated at the early stages of the cascade will be evaluated most favourably in short-duration assays, which is precisely the design used throughout this literature.

### 3.6. Methodological Quality of the Included Studies

**Figure 5:**
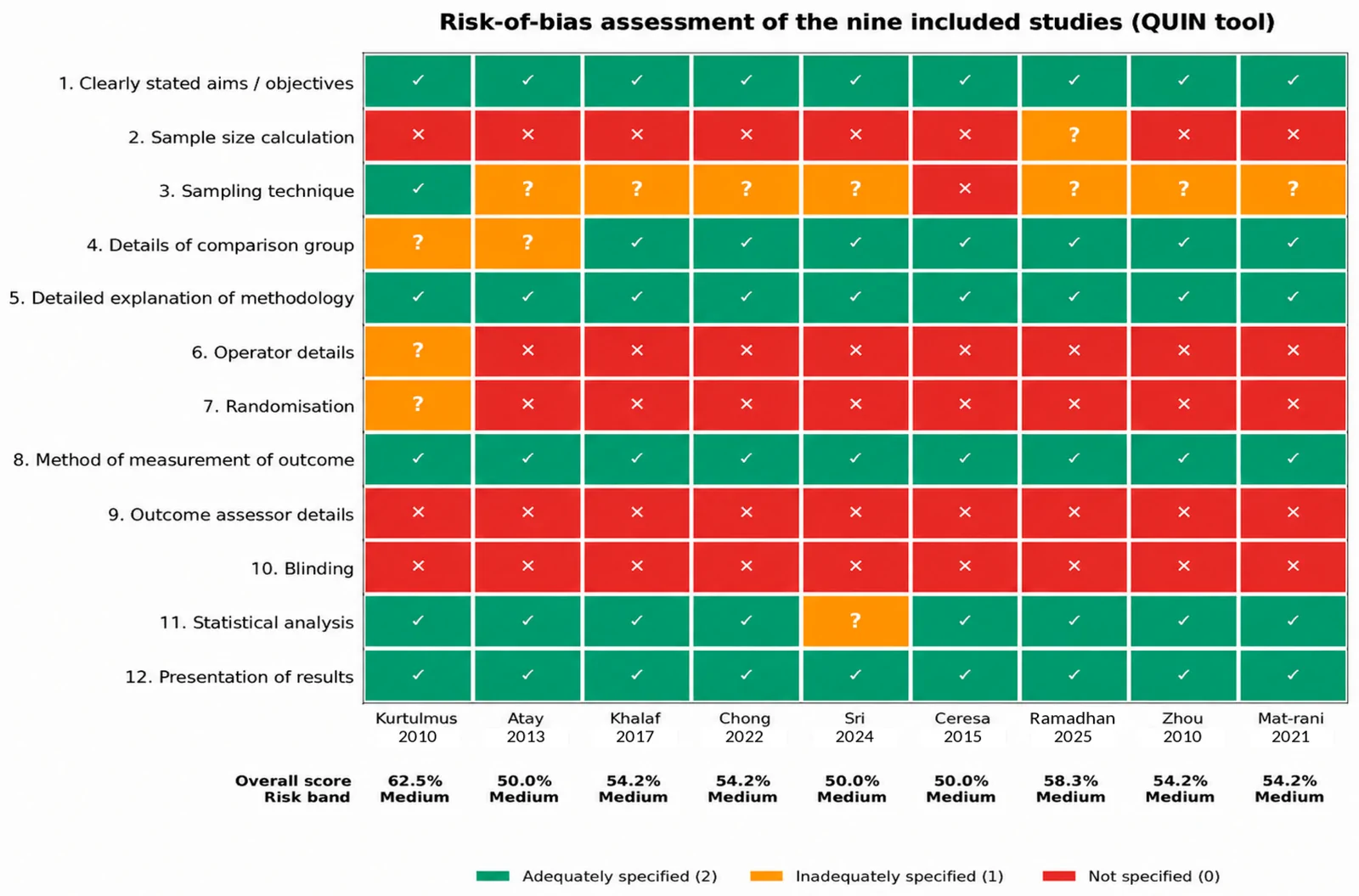
Risk-of-bias assessment of the nine included studies using the QUIN tool. Each of the twelve criteria was scored from the published full text as adequately specified (2), inadequately specified (1), or not specified (0). Overall scores are expressed as a percentage of the maximum twenty-four points and classified as low (>70%), medium (50–70%), or high (<50%) risk of bias. All nine studies fall within the medium band. Per-criterion justifications are given in Supplementary Table S1. Green cells marked with a tick (✓) indicate criteria that were adequately specified (2 points), orange cells marked with a question mark (?) indicate criteria that were inadequately specified (1 point), and red cells marked with a cross (×) indicate criteria that were not specified (0 points). The nine included studies were Kurtulmus et al. (2010) [18], Atay et al. (2013) [19], Khalaf et al. (2017) [20], Chong et al. (2022) [21], Sri et al. (2024) [22], Ramadhan and Abdul-Ameer (2025) [23], Zhou et al. (2010) [24], Mat-Rani et al. (2021) [25], and Ceresa et al. (2015) [26].

Risk of bias was appraised from the published full texts and is summarised in Figure 5. All nine studies fell within the medium-risk band, with overall scores ranging from 50.0 to 62.5%; none reached the low-risk threshold of 70%, and none fell below 50%. Kurtulmus et al. scored highest at 62.5%, and Ramadhan and Abdul-Ameer second at 58.3%, while Atay et al., Sri et al., and Ceresa et al. scored lowest at 50.0%.

Reporting was adequate across all nine studies for four domains: statement of aims, explanation of methodology, method of outcome measurement, and presentation of results. Statistical analysis was adequately specified in eight of nine; Sri et al. tabulated an F statistic and *p* value without naming the test applied or providing a statistical methods section [22].

Four domains were deficient throughout the evidence base and account for the uniform medium-risk classification. No study reported an a priori sample size calculation; the only design justification in this literature is the pilot study of Ramadhan and Abdul-Ameer, used to select the thymol concentrations carried into the main experiment [23]. No study identified the individual performing outcome assessment or reported their training or calibration. No study reported blinding of the assessor to group allocation. Only Kurtulmus et al. reported random allocation of specimens to groups, without describing how the sequence was generated [18]. Together, these four domains contribute six to eight points to the twenty-four-point obtainable score in every study.

The significance of this pattern is that the deficiency is one of reporting rather than of experimental conduct. Each domain requires a single additional sentence rather than any modification of laboratory practice, and their omission is what prevents an otherwise methodologically competent literature from being appraised as low risk.

### 3.7. The Conditioning-Film Gap

**Figure 6:**
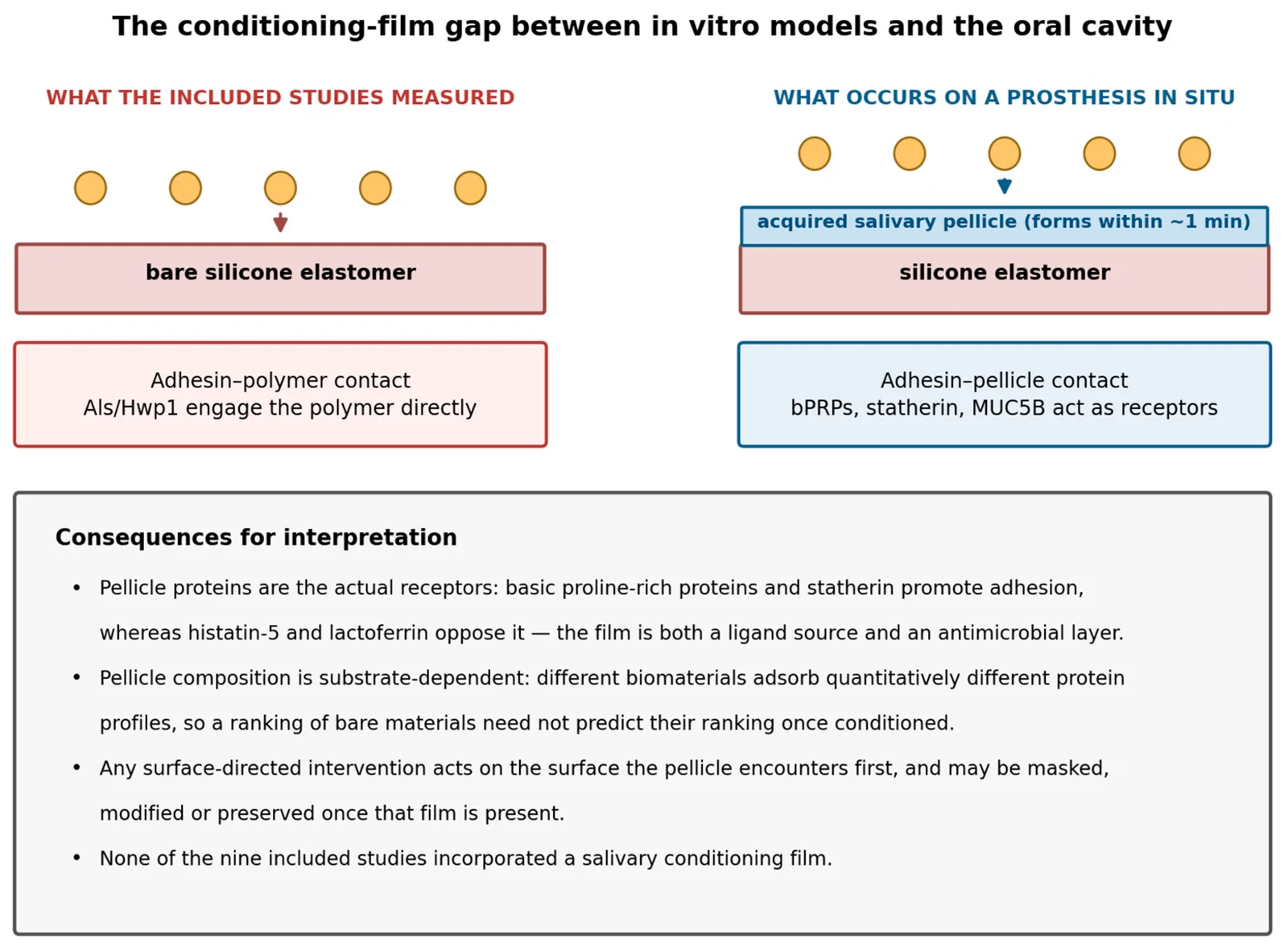
The conditioning-film gap between in vitro models and the oral cavity. Left: the experimental configuration used in all nine included studies, in which fungal adhesins contact the polymer directly. Right: the in situ configuration, in which an acquired salivary pellicle intervenes and supplies both adhesion receptors and antimicrobial peptides. Because pellicle composition varies with the underlying substrate, material rankings and intervention efficacies established on bare elastomer cannot be assumed to transfer. Red denotes the in vitro configuration evaluated across the included studies (left), and blue denotes the corresponding in situ configuration encountered in the oral cavity (right).

The most consequential limitation of the present evidence base is not any individual methodological weakness but a shared assumption. Most included studies evaluated maxillofacial silicone without prior conditioning by oral fluids, although the measured outcomes varied and not all directly assessed adhesion. Notably, Kurtulmus et al. examined silicone elastomers following exposure to simulated saliva and nasal secretion [18]. Nevertheless, systematic evaluation of the effects of an acquired salivary pellicle on *Candida* adhesion to maxillofacial silicone remains lacking (Figure 6).

An acquired salivary pellicle forms on oral surfaces within approximately one minute of exposure, and proteomic analyses have identified more than one hundred constituent proteins [55]. This film represents the surface that the organism encounters in the oral environment and is not biologically inert. Salivary proteins, including proline-rich proteins and statherin, can contribute to *Candida* adhesion to conditioned oral substrates [56,57], while salivary mucins can also interact directly with the organism [58]. Adhesion to saliva-coated substrates involves a partly distinct adhesin repertoire, including Bgl2p, Ecm33p, and Als1p [59]. Conversely, the pellicle may also exert protective effects, as histatin-5 inhibits adhesion [60], while lactoferrin and lactoperoxidase accumulate within the pellicle [61]. Critically, pellicle composition depends on the underlying substrate: different biomaterials adsorb quantitatively different protein profiles from the same saliva [62]. A ranking of bare elastomers by adhesion therefore need not survive conditioning, and a surface-directed intervention—a coating, a biosurfactant, an engineered change in wettability—acts on the surface that the pellicle encounters first and may be masked, modified, or preserved once that film is present. A review of the adjacent dental implant literature concludes that salivary pellicle proteins alter adhesion [63]. No equivalent study exists for maxillofacial silicone. This gap is particularly relevant to the antimicrobial and surface-modified silicones discussed above. None of the studies evaluating silver nanoparticle incorporation, chitosan reinforcement, thymol addition, parylene coating, or biosurfactant treatment applied salivary conditioning before testing, and there is accordingly no direct evidence showing whether an acquired pellicle masks, modifies, or preserves the activity of these interventions. Their reported efficacies were established on bare elastomer and cannot be assumed to persist once a conditioning film has formed.

## 4. Discussion

Interpretation of this evidence base through the molecular cascade summarised in Figure 2 yields three conclusions that do not follow from the materials-level reading, and each carries a distinct methodological consequence.

### 4.1. Surface Topography Constitutes a Morphogenetic Signal

The prevailing account of the roughness–adhesion relationship is essentially geometric: increased surface area affords more attachment sites, and topographic depressions shelter adherent cells from shear. Both effects undoubtedly operate. Neither, however, accommodates the evidence that *C. albicans* transduces topographic contact through a defined signalling pathway. Three lines of evidence indicate that surface geometry is instructive rather than merely permissive. Antagonists of stretch-activated and L-type calcium channels attenuate thigmotropic growth [27]; topographic responsiveness requires the cortical polarity marker Rsr1 [30]; and nanoscale features alone are sufficient to govern the yeast-to-hypha switch [32]. These three lines of evidence derive from the broader *C. albicans* literature on model substrates, not from maxillofacial silicone; the instructive role of topography proposed here is therefore a mechanistic hypothesis for this material rather than a demonstrated finding.

Two consequences follow. First, hyphal filaments furnish the mechanical scaffold of the mature biofilm, and the calcium-dependent pathway converges on the cyclic-AMP–protein kinase A axis that governs the Efg1–Tec1–Ume6 programme [29]. Topography therefore influences not only the quantity of adherent cells but also their developmental trajectory. Surface finishing may therefore be conceived as the withholding of a morphogenetic cue rather than the mere elimination of physical refuge. Second, transduction keyed to feature geometry commensurate with the sensing apparatus would produce threshold rather than linear behaviour. Such behaviour offers a parsimonious explanation for the loose correlation between arithmetic mean roughness and adhesion across the included studies and suggests that Ra alone may be insufficient to describe the biologically relevant features of surface topography. The biologically salient information plausibly resides instead in spatial parameters such as mean profile spacing, peak density, and characteristic feature wavelength, none of which is routinely reported. Their routine inclusion would permit the topography–response relationship to be modelled rather than asserted. This interpretation also has implications for the evaluation of antimicrobial surface coatings. The demonstration that *C. albicans* can survive on microcone-patterned substrates despite the effectiveness of antifungal coatings on planar controls [33] suggests that surface topography may influence the efficacy of antimicrobial surface treatments. Therefore, antimicrobial efficacy demonstrated on polished specimens should not necessarily be assumed to translate directly to milled or additively manufactured surfaces. This consideration is particularly relevant to maxillofacial prosthetic materials, given the increasing adoption of digital fabrication workflows. A related caveat concerns the attribution of adhesion effects to individual surface properties. Surface roughness, porosity, wettability, and surface chemistry are interrelated and may vary concurrently; for example, polishing can modify both surface topography and surface energy, while moulding conditions or surface coatings may alter surface chemistry alongside roughness. Because the included studies did not consistently characterise these parameters, either individually or in combination, the reported differences in *Candida albicans* adhesion cannot be attributed confidently to a single surface property in isolation.

### 4.2. Reported Efficacies Are Not Commensurable

Ranking interventions by percentage reduction presupposes a comparability that the target mapping in Figure 4 does not support. An interfacial agent operating before attachment, a transcriptional agent compromising cell-wall integrity, and a pro-apoptotic agent acting on mitochondrial function are differentially advantaged by particular assay configurations. Short exposure intervals favour agents acting at early cascade stages; endpoints indexed to metabolic activity favour fungicidal over anti-adhesive mechanisms; and endpoints indexed to colony counts favour the converse. Reported efficacy is therefore partly dependent on assay design and may not fully represent the intrinsic activity of the agent.

This observation reframes the heterogeneity that has been noted repeatedly in this field. The inconsistency between studies is not principally attributable to methodological imprecision and would not be resolved by more sophisticated statistical treatment or by standardisation of outcome units alone. It reflects genuine mechanistic diversity among agents that have been grouped by a shared endpoint. Meaningful comparison requires that agents be evaluated at the cascade stage at which they act, or alternatively that a common assay be adopted which interrogates several stages concurrently.

The mapping additionally identifies an absence. No agent evaluated on maxillofacial silicone engages matrix assembly or biofilm-phase tolerance, notwithstanding that the β-1,3-glucan matrix is the important determinant of antifungal tolerance in *Candida* biofilms [50,51,54], and that tolerance mechanisms themselves shift from efflux-mediated to sterol-mediated with biofilm maturation [53,54]. Agents concentrated at early cascade stages will be evaluated most favourably under short-duration assay conditions, which are precisely those employed throughout this literature. The apparent efficacy of the current intervention repertoire may therefore be partly an artefact of the interval over which it has been assessed.

### 4.3. The Evidence Base Characterises a Surface That Does Not Occur Clinically

The most consequential limitation of this literature is not a deficiency of any individual study, but the limited evaluation of salivary conditioning: most included studies evaluated maxillofacial silicone without prior conditioning by oral fluids, while Kurtulmus et al. examined silicone following exposure to simulated saliva and nasal secretion [18]. In situ, an acquired salivary pellicle forms within approximately one minute of exposure [55] and constitutes the surface that the organism actually encounters. This film is not inert: basic proline-rich proteins and statherin function as adhesion receptors [56,57,58], adhesion to conditioned substrates engages a partly distinct adhesin repertoire [59], and the same film delivers opposing antimicrobial activity through histatin-5, lactoferrin, and lactoperoxidase [60,61].

Critically, pellicle composition is substrate-dependent, with different biomaterials adsorbing quantitatively distinct protein profiles from identical saliva [64]. Two inferences follow. A ranking of bare elastomers by adhesion need not be preserved after conditioning, since the conditioning film is itself a function of the material beneath it. Surface-directed interventions face the same difficulty. A coating, a biosurfactant, or an engineered alteration of wettability modifies the surface that the pellicle encounters first, so its effect may be masked, altered, or preserved once the film is established. Which of these is obtained cannot presently be determined for any intervention evaluated on maxillofacial silicone, since the question has not been examined. The nearest precedent, a narrative review of the dental implant literature, concludes that salivary pellicle proteins can alter adhesion [63]. Direct evidence for maxillofacial silicone is absent: no included study conditioned antimicrobial-modified or surface-modified specimens with saliva before testing, so the effect of the pellicle on these interventions remains unknown.

This consideration bounds rather than invalidates the existing evidence. Characterisation of adhesin–polymer interaction on defined surfaces is legitimate and mechanistically informative. What it does not support is inference to clinical performance, and the prevailing practice of extrapolating from bare-surface colony counts to anticipated prosthesis longevity is not warranted by the available designs.

### 4.4. Implications for the Design of Future Studies

Three modifications would materially advance this field, in ascending order of resource requirement. Adequate surface characterisation is the least demanding: spatial roughness parameters, contact angle with derived surface free energy, and representative micrographs should accompany every adhesion measurement, since the topography–response relationship cannot otherwise be modelled. Incorporation of a salivary conditioning film offers the greatest return relative to cost. Pooled stimulated whole saliva, a defined incubation interval, and paired bare and conditioned arms would establish, within a single experiment, how far the existing literature transfers to the clinical substrate. A targeted transcriptional readout is the most demanding addition but yields the greatest interpretive gain. Quantitative PCR of ALS1, ALS3, and HWP1 for adhesion; UME6 and NRG1 for morphogenetic commitment; ZAP1 for matrix regulation; and CDR1 and MDR1 for tolerance would separate an agent that prevents attachment from one that suppresses the hyphal programme, and both from one that is simply fungicidal. That distinction is inaccessible to colony enumeration yet determines how an agent should be deployed clinically.

Finally, the risk-of-bias appraisal identifies four domains in which reporting failed uniformly: sample size justification, operator identification, assessor identification, and blinding. These are deficiencies of reporting rather than of conduct, remediable in a few sentences, and their persistence is why no study in this field currently achieves a low risk-of-bias classification.

## 5. Limitations

This review has a deliberate methodological asymmetry: the appraised evidence base was assembled systematically, whereas the molecular framework used to interpret it was assembled by targeted searching and selected for mechanistic relevance. Relevant molecular work may therefore have been omitted, and the framework should be read as an interpretive structure rather than an exhaustive synthesis.

The evidence base itself is small and heterogeneous. Nine studies, most with fewer than fifty specimens and none reporting a sample size calculation, evaluated different materials with different preparation protocols, inocula, exposure durations, control definitions, and outcome measures. Outcomes reported as colony-forming units, cells per unit area, tetrazolium reduction, and inhibition-zone diameter are not commensurable, and no quantitative pooling was attempted; percentage reductions cited here are interpretable only within their source study. Restriction of eligibility to English-language publications and the preferential publication of positive antimicrobial findings both plausibly inflate apparent efficacy. One included study departed from eligibility criterion (ii): Ceresa et al. [26] evaluated a medical-grade silicone of the type used in voice prostheses rather than a designated maxillofacial elastomer. It was retained because it is the only evaluation of a biosurfactant intervention on a clinically adjacent silicone, but its findings should be read with that proviso.

Risk-of-bias appraisal placed every included study in the medium band, and the absence of reported randomisation, assessor identification, and blinding throughout means that measurement bias cannot be excluded for any study in this review. The molecular mechanisms discussed were, with few exceptions, established in planktonic culture or on model substrates such as polystyrene and hydroxyapatite rather than on maxillofacial silicone. Their operation on these specific elastomers is inferred, not demonstrated. None of the included studies reported any molecular readout, so the mapping in Figure 4 is a synthesis across studies rather than a direct observation. Finally, all included studies were in vitro, and none evaluated durability, mechanical consequence, or biocompatibility over an interval approaching the service life of a prosthesis. In addition, surface properties were not characterised uniformly across the included studies, and roughness, porosity, wettability, and surface chemistry are interdependent rather than independent variables; differences in *Candida* adhesion between materials therefore cannot always be attributed to a single surface parameter.

## 6. Conclusions

Interpreted molecularly, the in vitro literature on *Candida albicans* adhesion to maxillofacial silicone elastomers supports three conclusions. Evidence from broader *C. albicans* studies indicates that topographic contact can activate calcium-dependent mechanosensory pathways associated with hyphal commitment. Surface finishing may therefore be understood as withholding a morphogenetic cue, and spatial rather than amplitude roughness parameters should be reported. The antimicrobial strategies evaluated on these materials engage at least four distinct molecular stages and are consequently not interchangeable, which explains why their reported efficacies resist meaningful comparison and identifies matrix assembly and biofilm-phase tolerance as stages no tested agent currently addresses. Most included studies evaluated silicone without salivary conditioning, whereas the clinical substrate is an acquired salivary pellicle that supplies both adhesion receptors and antimicrobial peptides and whose composition depends on the material beneath it. The evidence base is therefore mechanistically informative but describes a surface that does not occur in situ. Incorporating a salivary conditioning film and a targeted gene-expression readout into otherwise conventional adhesion assays would address both limitations at a modest cost and is the most direct route to evidence capable of supporting clinical recommendations.

## Data Availability

No new data were created or analysed in this study. Data sharing is not applicable to this article. All data supporting the findings are available within the manuscript and its referenced sources.

## Supplementary Materials

The following supporting information can be downloaded at https://www.mdpi.com/article/10.3390/ijms27209031/s1.

## Abbreviations

The following abbreviations are used in this manuscript:

AFR	Amyloid-forming region
Al_2_O_3_	Aluminium oxide
Als	Agglutinin-like sequence
cAMP	Cyclic adenosine monophosphate
CFU	Colony-forming unit
CSH	Cell-surface hydrophobicity
OSF	Open Science Framework
PBC	Peptide-binding cavity
PKA	Protein kinase A
QUIN	Quality Assessment Tool for In Vitro Studies in Dentistry
Ra	Arithmetic mean surface roughness
ROS	Reactive oxygen species
SEM	Scanning electron microscopy
TiO_2_	Titanium dioxide
