# Supplementary material for: Molecular Mechanisms of Candida albicans Adhesion to Maxillofacial Silicone Elastomers: Integrating Candidate Molecular Pathways with Material Surface Properties
Source: Int J Mol Sci. 2026 Oct 12;27(20):9031. doi: 10.3390/ijms27209031 (PMC13705479; doi:10.3390/ijms27209031)
Supplement: Supplementary file 1 [file ijms-27-09031-s001.zip › ijms-4456021-supplementary.pdf]

Supplementary Material

Table S1. Risk-of-bias assessment of the nine included studies using the QUIN tool: per-criterion basis for scoring

Scoring follows the QUIN tool for in vitro studies in dentistry. Each of twelve criteria was scored as adequately specified (2), inadequately specified (1), or not specified (0). The overall score is the sum expressed as a percentage of the maximum 24 points, classified as low (>70%), medium (50-70%) or high (<50%) risk of bias. Criteria 1, 5, 8 and 12 were adequately specified in all nine studies and are therefore not itemised below; criteria are listed individually only where the score was other than 2 or where the basis merits statement. All scores were assigned from the published full texts.

Summary of overall scores: Kurtulmus 62.5%, Ramadhan 58.3%, Khalaf 54.2%, Chong 54.2%, Zhou 54.2%, Mat-Rani 54.2%, Atay 50.0%, Sri 50.0%, Ceresa 50.0%. All nine studies fall within the medium risk-of-bias band (range 50.0-62.5%); none reached the low risk threshold and none fell below it.

Study	Criterion	Basis for score in the source text	Score
Kurtulmus 2010 [20]	2	No a priori sample size calculation or power analysis reported. Specimen numbers are stated (N = 48; n = 16 per group; n = 8 per subgroup) but numbers alone do not satisfy this criterion.	**0**
	3	"They were then randomly divided into two subgroups (n = 8)." Allocation of specimens to groups is explicitly described.	**2**
	4	Comparison is between materials and between exposure media; no independent untreated reference condition is defined.	**1**
	6	"All specimens were fabricated by one operator at 20-25 °C room temperature." Operator identified as a single individual, but no training, calibration or experience data.	**1**
	7	Random division stated but the method of randomisation (sequence generation) is not described.	**1**
	9,10	No information on who performed colony assessment; no blinding reported.	**0**
Atay 2013 [21]	2	No sample size calculation. Specimen numbers stated (n = 8 per group, N = 24).	**0**
	3	Group sizes given but no description of how specimens were allocated.	**1**
	4	Three silicone materials compared against one another; no independent control condition.	**1**
	6,7,9,10	No operator, randomisation, assessor or blinding information.	**0**
	11	Kruskal-Wallis and Mann-Whitney U tests named; significance threshold stated ($P < 0.01$).	**2**
Khalaf 2017 [22]	2	No sample size calculation. Total stated as N = 35.	**0**
	4	Coated versus uncoated gypsum mould surfaces; comparison condition clearly defined.	**2**
	11	t-test, one-way ANOVA and post hoc tests named; $p < 0.05$ threshold stated.	**2**
	6,7,9,10	Not reported.	**0**
Chong 2022 [18]	2	No sample size calculation. 180 disks reported; assays in triplicate.	**0**

Study	Criterion	Basis for score in the source text	Score
	4	Negative control (silicone without AgNP) defined; concentrations 0.1% and 0.5% compared against it.	**2**
	11	Two-way ANOVA, paired t test and repeated-measures ANOVA named; $P < .001$ reported.	**2**
	6,7,9,10	Not reported.	**0**
Sri 2024 [44]	2	No sample size calculation. 80 specimens in four groups of 20.	**0**
	4	Group C1 defined as control silicone without chitosan.	**2**
	11	F score and P value (< 0.0001) tabulated, but no statistical methods section and the test is never named.	**1**
	6,7,9,10	Not reported.	**0**
Ceresa 2015 [49]	2	No sample size calculation and no total specimen number stated; assays described as performed in triplicate and repeated three times.	**0**
	3	No description of how silicone disks were allocated to test and control conditions.	**0**
	4	Untreated silicone disks serve as control; pre-coating and co-incubation conditions defined.	**2**
	11	Analysis performed in R; means and standard deviations reported throughout.	**2**
	6,7,9,10	Not reported.	**0**
Ramadhan 2025 [48]	2	No formal power calculation, but a pilot study was conducted and used to select the 0.75 and 1 wt% concentrations for the main study, providing partial justification for the design.	**1**
	4	Four groups defined including a negative control (no additive) and a positive control (1.4 wt% nystatin).	**2**
	11	ANOVA with post hoc multiple comparison tests named; SPSS used; significance set at $P < 0.05$.	**2**
	6,7,9,10	Not reported.	**0**
Zhou 2010 [23]	2	No sample size calculation. $n = 8$ reported for contact angle measurement.	**0**
	4	Uncoated silicone elastomer and denture base resin serve as controls against parylene-coated specimens.	**2**
	11	Analyses performed in SPSS 15.0.	**2**
	6,7,9,10	Not reported.	**0**
Mat-Rani 2021 [19]	2	No sample size calculation. 42 specimens ($n = 21$ per material) reported.	**0**
	4	Nystatin 20% positive control and medium-only control defined.	**2**
	11	Regression coefficient and post hoc Tukey HSD named; exact P values reported ($P = 0.031$, $P = 0.99$, $P = 0.302$).	**2**
	6,7,9,10	Not reported.	**0**

Domains of consistent deficiency

Four QUIN domains were deficient across the entire evidence base. No study reported an a priori sample size calculation, and only Ramadhan and Abdul-Ameer provided any design justification, in the form of a pilot study used to select test concentrations. No study reported the identity, training or calibration of the person performing outcome assessment. No study reported blinding of the assessor to group allocation. Only Kurtulmus et al. reported random allocation of specimens to groups, and did not describe the method by which the sequence was generated.

These four domains together account for 6 to 8 of the 24 available points in every study and are the principal reason that no study achieved a low risk-of-bias classification. They are also, notably, the domains most readily remedied: each requires only a sentence of additional reporting rather than any change to experimental practice.

Supplementary Material

Table S2. PRISMA-ScR checklist

Reporting follows the Preferred Reporting Items for Systematic Reviews and Meta-Analyses extension for Scoping Reviews (PRISMA-ScR; Tricco et al., Ann. Intern. Med. 2018, 169, 467–473). Items 12 and 16 are designated optional within PRISMA-ScR; both were completed for this review, since critical appraisal of the included in vitro studies formed part of the review objectives.

Item	Section / topic	How addressed in this review	Location
**TITLE**			
1	Title	Identifies the report as a scoping review.	Title page
**ABSTRACT**			
2	Structured summary	Structured summary provides background, objectives, eligibility criteria, sources of evidence, charting methods, results and conclusions relating to the review question.	Abstract
**INTRODUCTION**			
3	Rationale	Describes the rationale for the review in the context of what is already known, and explains why the review question lends itself to a scoping review approach.	Section 1, paragraphs 2–4
4	Objectives	States the question addressed with reference to the Population–Concept–Context framework, and states the three review objectives explicitly.	Section 1, final paragraph; Section 2.2
**METHODS**			
5	Protocol and registration	States that a protocol was prepared, gives the registry name and registration number, and confirms that no post-registration amendments were made.	Section 2.1
6	Eligibility criteria	Specifies characteristics of sources of evidence used as eligibility criteria, with inclusion and exclusion criteria enumerated separately and rationale given for the exclusion of in vivo designs.	Section 2.4
7	Information sources	Describes all information sources, gives the vendor and location of each database, states the date range covered and the date the final search was executed, and reports hand-searching of reference lists.	Section 2.3

Item	Section / topic	How addressed in this review	Location
**8**	Search	Presents the full search strategy, including the three Boolean concept blocks and all terms within each; the complete string as executed in each database is reproduced in Supplementary Table S3.	Section 2.3; Supplementary Table S3
**9**	Selection of sources of evidence	States the process for selecting sources of evidence, including the number of reviewers screening independently and the method of resolving disagreements.	Section 2.5
**10**	Data charting process	Describes the method of charting data, states that the form was developed a priori and piloted, gives the number of reviewers charting and verifying, and states that unreported items were recorded as "not stated" rather than inferred.	Section 2.6
**11**	Data items	Lists and defines all fifteen variables for which data were sought.	Section 2.6
**12**	Critical appraisal of individual sources of evidence	Describes the QUIN instrument, lists all twelve criteria, gives the scoring rule and the percentage formula, states the risk-of-bias thresholds, and states that appraisal contextualised rather than weighted the synthesis.	Section 2.7
**13**	Synthesis of results	Describes the narrative synthesis approach and states the reason quantitative pooling was not attempted.	Section 2.8
**RESULTS**			
**14**	Selection of sources of evidence	Gives numbers of sources screened, assessed for eligibility and included, with reasons for exclusion at each stage, presented in a flow diagram.	Section 3.1; Figure 1
**15**	Characteristics of sources of evidence	Presents characteristics of each source, including materials evaluated, specimen numbers and interventions tested.	Section 3.1; Sections 3.2–3.5
**16**	Critical appraisal within sources of evidence	Presents the critical appraisal of each source, with per-criterion scores, overall percentages and risk-of-bias classification.	Section 3.6; Figure 6; Supplementary Table S1
**17**	Results of individual sources of evidence	Presents the relevant data charted from each source, related to the review objectives.	Sections 3.2–3.5, 3.7
**18**	Synthesis of results	Summarises and synthesises the results as they relate to the review question and	Sections 3.2–3.7; Figures 2–5

Item	Section / topic	How addressed in this review	Location
		objectives, organised by cascade stage.	
**DISCUSSION**			
**19**	Summary of evidence	Summarises the main results, links them to the review objectives, and considers their relevance to material developers, clinicians and researchers.	Sections 4.1–4.4
**20**	Limitations	Discusses the limitations of the scoping review process, including the deliberate methodological asymmetry between the two identification arms, the non-commensurability of outcome measures, and the derivation of mechanistic evidence from other substrates.	Section 5
**21**	Conclusions	Provides a general interpretation of the results with respect to the review question and objectives, and identifies the implications for further research.	Section 6
**FUNDING**			
**22**	Funding	Describes sources of funding for the review and the role of funders.	Funding statement

Supplementary Material

Table S3. Search strategy as executed in each database

The search combined three concept blocks with the Boolean operator AND, with terms within each block combined with OR. Block 1 specified the organism, Block 2 the substrate, and Block 3 the outcome or intervention. Truncation is indicated by an asterisk. The strategy was adapted to the syntax and field-tag conventions of each database as set out below.

S3.1. Concept blocks

Block	Terms
Block 1 — organism	"Candida albicans", Candida, fungal
Block 2 — substrate	"Maxillofacial silicone", "silicone elastomer*", "maxillofacial prosthes*", "facial prosthes*"
Block 3 — outcome or intervention	adhesion, adherence, biofilm*, colonis*, coloniz*, antifungal, antimicrobial, nanoparticle*

S3.2. PubMed (National Library of Medicine, Bethesda, MD, USA)

```
(((("Candida albicans"[Title/Abstract]) ) OR (Candida[Title/Abstract])) OR
(fungal[Title/Abstract])) AND (((("maxillofacial silicone"[Title/Abstract]) OR ("silicone
elastomer*"[Title/Abstract])) OR ("maxillofacial prosthes*"[Title/Abstract])) OR ("facial
prosthes*"[Title/Abstract]))) AND ((((((((adhesion[Title/Abstract]) OR (adherence[Title/Abstract]))
OR (biofilm*[Title/Abstract])) OR (colonis*[Title/Abstract])) OR (coloniz*[Title/Abstract])) OR
(antifungal[Title/Abstract])) OR (antimicrobial[Title/Abstract])) OR
(nanoparticle*[Title/Abstract]))
```

Records retrieved: 56

S3.3. Scopus (Elsevier, Amsterdam, The Netherlands)

```
TITLE-ABS-KEY("Candida albicans" OR candida OR fungal)
  AND TITLE-ABS-KEY("maxillofacial silicone" OR "silicone elastomer*"
       OR "maxillofacial prosthes*" OR "facial prosthes*")
  AND TITLE-ABS-KEY(adhesion OR adherence OR biofilm* OR colonis* OR coloniz*
       OR antifungal OR antimicrobial OR nanoparticle*)
```

Records retrieved: 128

S3.4. ScienceDirect (Elsevier, Amsterdam, The Netherlands)

ScienceDirect advanced search permits a maximum of eight Boolean connectors per query and does not support wildcard truncation. The strategy was therefore simplified for this database, and the reduced sensitivity is acknowledged as a limitation of the search.

```
Title, abstract, keywords:
("Candida albicans") AND ("maxillofacial silicone" OR "silicone elastomer")
  AND (adhesion OR biofilm OR antifungal OR antimicrobial)
```

Records retrieved: 12

S3.5. Summary of yield

Source	Records	Date searched	Notes
PubMed	[56]	31 March 2026	–
Scopus	[128]	31 March 2026	–

Source	Records	Date searched	Notes
ScienceDirect	[12]	31 March 2026	Boolean operators limited to eight; no truncation
Total database records before de-duplication	196		As reported in Figure 1
Duplicates removed	[44]		
Total after de-duplication	[152]		Carried to title and abstract screening
Hand-searching of reference lists	12	31 March 2026	Reference lists of the included articles; performed after study selection
